# Molecular evolution of synonymous codon usage in *Populus*

**DOI:** 10.1186/1471-2148-8-307

**Published:** 2008-11-04

**Authors:** Pär K Ingvarsson

**Affiliations:** 1 Umeå Plant Science Centre, Department of Ecology and Environmental Science, Umeå University, SE90187 Umeå, Sweden

## Abstract

**Background:**

Evolution of synonymous codon usage is thought to be determined by a balance between mutation, genetic drift and natural selection on translational efficiency. However, natural selection on codon usage is considered to be a weak evolutionary force and selection on codon usage is expected to be strongest in species with large effective population sizes.

**Results:**

I examined the evolution of synonymous codons using EST data from five species of *Populus*. Data on relative synonymous codon usage in genes with high and low gene expression were used to identify 25 codons from 18 different amino acids that were deemed to be preferred codons across all five species. All five species show significant correlations between codon bias and gene expression, independent of base composition, thus indicating that translational selection has shaped synonymous codon usage. Using a set of 158 orthologous genes I detected an excess of unpreferred to preferred (*U *→ *P*) mutations in two lineages, *P. tremula *and *P. deltoides*. Maximum likelihood estimates of the strength of selection acting on synonymous codons was also significantly greater than zero in *P. tremula*, with the ML estimate of 4*N*_*e*_*s *= 0.720.

**Conclusion:**

The data is consistent with weak selection on preferred codons in all five species. There is also evidence suggesting that selection on synonymous codons has increased in *P. tremula*. Although the reasons for the increase in selection on codon usage in the *P. tremula *lineage are not clear, one possible explanation is an increase in the effective population size in *P. tremula*.

## Background

Codon bias, the preferential use of subset of synonymous codons, has been documented in a wide variety of organisms, from prokaryotes, to unicellular and multicellular eukaryotes [1-3]. While codon bias appears to be almost universal, the magnitude of codon bias largely depends on the effective population size, with codon bias being higher in species with larger effective population sizes [4]. Evolution of synonymous codon usage is a process where natural selection is sufficiently weak (*N*_*s *_~ 1) that the outcome is influenced by both selection, mutation and genetic drift [1,2]. At the same time, synonymous changes within and between species are sufficiently common that abundant data is available for testing evolutionary hypotheses of synonymous codon usage, explaining why much attention has been directed to understanding the relative contributions of mutation, genetic drift and natural selection to the patterns of codon bias seen within and between species.

The most common model of synonymous codon usage, the "major codon preference model", assumes that one or a few synonymous codons are preferentially used in genes with high codon bias [3,5]. Such codons are usually termed "preferred codons" and often end in either C of G. In most cases preferred codons correspond to the most abundant tRNAs from each codon family and codon bias is therefore thought to have arisen in response to natural selection favoring transcriptional accuracy or efficiency [3]. Under the assumption of a balance between weak selection and genetic drift, the expected proportion of sites fixed for the preferred codon is

(1) $$E[F_{op}]=\frac{1}{1+(\mu /\nu )e^{-4Ns}}$$

where *μ *and *ν *are the mutation rates from and to preferred codons, respectively, and 4*N s *is the scaled selection coefficient acting on preferred codons [6,7]. Equation (1) suggest that changes in evolutionary parameters, such as population size or mutation rate, can have large effects on the evolution of synonymous codon usage even in closely related species. While major shifts in codon preferences are unlikely between closely related species, changes in evolutionary parameters, particular in effective population sizes, are relatively common even over short evolutionary time scales [5]. Population size changes are expected to affect codon usage across all synonymous codon families and results in enhanced codon bias following a population size increase and an erosion of codon bias after a population size decline [5]. For instance, several studies have documented reductions in codon bias in *Drosophila melanogaster *and an increased fixation rate of unpreferred codons compared to the closely related species *D. simulans *and this has been attributed to a reduction in the effective population size of *D. melanogaster *and a relaxation of selection on codon bias [8-10]. Reductions in codon bias, likely driven by reductions in effective population sizes, have also been documented in *D. miranda *[11] and several species from the *D. virils *group [12]. Mating system is another potential factor affecting effective population sizes. For instance, self-fertilization is associated with a reduction in the effective population size [13] and *Caenorhabditis *species which have experienced a transition from obligately outcrossing to selfing show a concomitant decrease in codon bias [14].

I have recently shown that patterns of synonymous codon usage in the long-lived tree *Populus tremula *is consistent with weak natural selection acting on translational efficiency as evidenced by a strong positive correlation between gene expression and codon bias [15]. In this paper I extend my earlier studies of codon bias in *P. tremula *to five species covering most of the phylogenetic diversity found within the genus *Populus*. Using a greatly expanded set of genes and species allows for more thorough studies of the molecular evolution of synonymous codon usage across lineages with the hope of answering questions pertaining to the stability of evolutionary parameters, such as mutation rates, population sizes and patterns of natural selection.

## Results

### Identification of optimal codons

**Figure 1:**
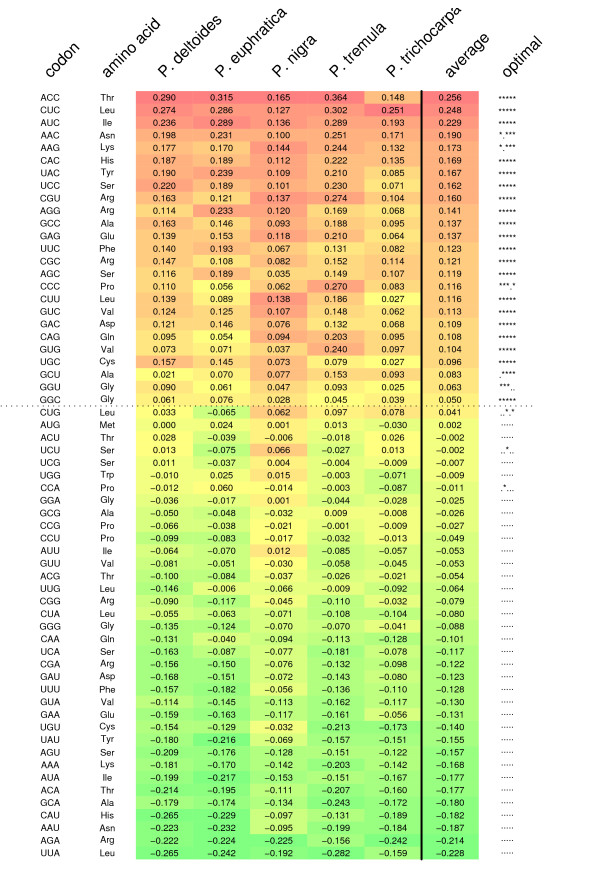
**Difference in relative synonymous codon usage (RSCU) across codons between genes with high and low gene expression.** Codons are sorted after average ΔRSCU. The right-most column indicate codons with significantly increased usage in high-expression genes as determined by a *t*-test. Each * denotes a species for which the *t*-test was significant, in the same order as they are listed in the Figure. Codons above the horizontal dotted lines were used to designed optimal codons and were used to calculate frequencies of optimal codon usage (*F*_*op*_) in all species. The colors in the figure indicate the gradient of ΔRSCU values, from the most positive(orange) to the most negative (green).

Optimal codons were identified for all five species based on differences in relative synonymous codon usage (ΔRSCU) between genes with high and low levels of gene expression (Figure 1). Using this approach I identified 24 codons showing significant ΔRSCU values between low and high expression genes in four or more species. An additional four codons showed ΔRSCU values that were significant in three or few species. Some of the codons that do not show significant differences between high and low expression genes in all species nevertheless have positive ΔRSCU values in all species (e.g. AAC, AAG and GGU). These are likely optimal codons (as evidenced by their positive ΔRSCU values) but where power might be too low to achieve statistical significance in one or a few species. On the other hand, some codons show reversal of ΔRSCU between high and low expression genes across species (e.g. CUG or UCU). Whether these codons truly represent differences in codon preferences between species or simply represent statistical artifacts is not clear. Based on the results from the ΔRSCU analysis, I identified 25 codons from 18 different amino acids that were used to calculate the frequency of optimal codon usage in all genes of the different species (Figure 1). Most, but not all, of these codons end with either a C or a G, as have been observed in many other species [2].

In a recent study I estimated optimal codons in *P. tremula *based on correspondence analysis of codon usage in 558 different genes using the program codonw [15]. The results presented here are both based on more species (five vs. one) and substantially more genes from each species (> 4500 genes per species). Nevertheless there is a large degree of overlap between optimal codons I identified in *P. tremula *[15] and those identified in this study. In fact, the 11 codons identified as optimal in *P. tremula *[15] represents a subset of the 25 codons identified in this study. The greater number of optimal codons identified here is likely the result of a greater power to identify subtle differences in codon usage between high and low expression genes due to the substantially larger data set used in this study.

### Factors explaining variation in synonymous codon usage

**Figure 2:**
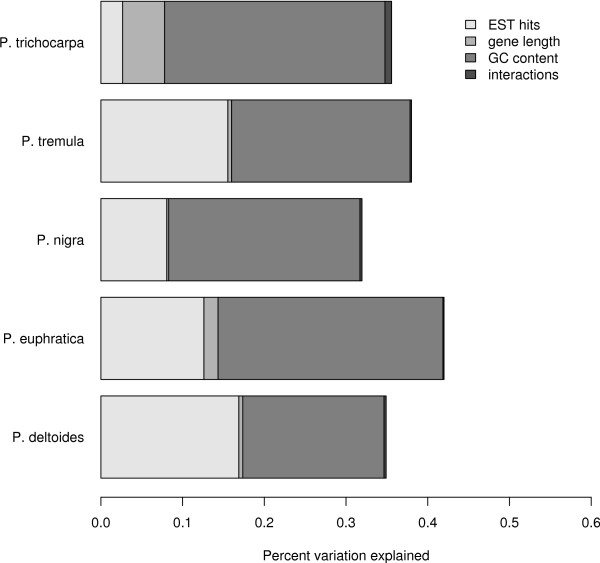
Proportion of variation in the frequency of optimal codon usage explained by gene expression, gene length and base composition at synonymous sites.

There is a significant positive correlation between gene expression, measured as the number of hits from the EST data, and codon bias, measured as the frequency of optimal codon usage (Table 1). These correlations are independent of base composition and sequence lengths and are consistent with natural selection on translational accuracy and/or efficiency having shaped codon usage. There are large differences among species in the amount of variation in codon usage explained by gene expression, ranging from 2.6% in *P. trichocarpa *to 16.9% in *P. deltoides *(Figure 2). The base composition of sequences also explain a sizable fraction of the variation in codon usage, ranging from 17.2% in *P. deltoides *to 28.5% in *P. euphratica*. Interestingly, gene length appears to have little effect on codon usage and so do interactions between gene expression, base composition and gene length (Figure 2). These results differ to some degree from those I obtained in my earlier study of codon usage in *P. tremula *[15]. The main difference is that base composition explain a large fraction of the variation in codon usage in the current data set whereas the effect of gene length is weak and non-significant. The main conclusion remains the same, however, namely that codon usage in all species is, to a large degree, shaped by translational selection. As an overall index of codon usage bias I calculated the average of all positive ΔRSCU values ($\bar{\Delta {\text{RSCU}}_{+}}$) [16]. There is a strong correspondence between $\bar{\Delta {\text{RSCU}}_{+}}$ values and the strength of the association between gene expression and codon usage across species (Table 1).

**Table 1:** Number of unique transcripts and an index of codon usage bias for five different species of *Populus*.

Species	No. genes	cor(EST hits, *F*_*op*_)	$\bar{\Delta {\text{RSCU}}_{+}}$
*P. deltoides*	5142	0.273 ***	0.145
*P. euphratica*	4687	0.248 ***	0.149
*P. nigra*	15386	0.210 ***	0.094
*P. tremula*	6534	0.268 ***	0.193
*P. trichocarpa*	13081	0.207 ***	0.097

See text for further detail.

### Phylogeny of the five species

**Figure 3:**
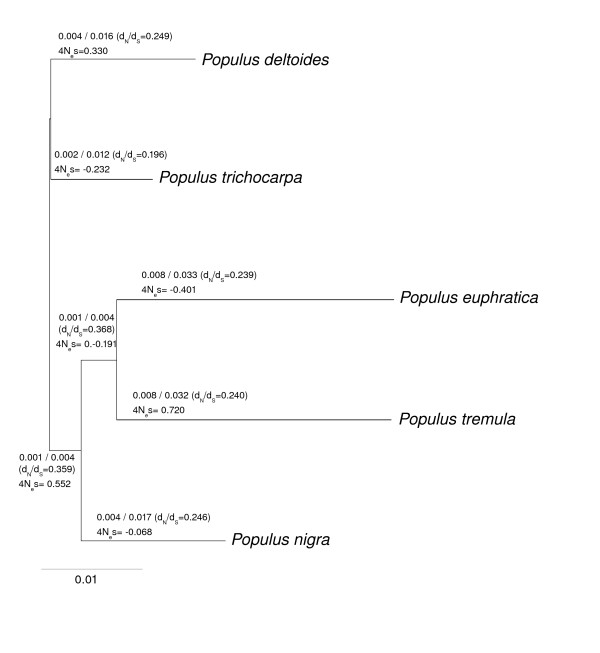
**Unrooted tree representing the phylogenetic relationship between the five species.** ML estimates of non-synonymous (*d*_*N*_) and synonymous (*d*_*S*_) substitution rates, *d*_*N*_*/d*_*S *_ratios (in parentheses) and the maximum likelihood estimates of selection acting on preferred codons (4*N*_*e*_*s*) are shown above each branch and are calculated from the concatenated data set of 158 genes. See text for further details. Branch lengths are proportional to synonymous substitution rates. All branches have 100% bootstrap support.

Putative one-to-one orthologous genes were identified from the five different species using the program orthoMCL [17]. This search identified a total of 158 orthologous groups that had all five species represented. The average length of the aligned regions was 576 bp and the concatenated data set contained a total of 71361 bps of coding sequences per species. The concatenated data set was used to estimate the phylogeny of the five species and the phylogenetic tree obtained is depicted in Figure 3. The support for the tree topology in Figure 3 is strong, with 100% bootstrap support for all branches when using the concatenated data. This tree is also consistent across different substitution models and tree inference methods (NJ, ML or parsimony), all of which yielded the same tree (data not shown). The tree in Figure 3 is also consistent with earlier phylogenetic studies of the genus *Populus *and the Salicaceae family using both morphological data [18] or cpDNA and ITS data [19,20]. However, because of the low sequence divergence among the species and the stochastic nature of the coalescent, there are possibilities that trees estimated for individual genes are not congruent. To test this, I estimated the likelihood for a number of variants on the trees depicted in Figure 3 for each of the 158 genes separately. The tree in Figure 3 was found to be the most likely tree for approximately 57% of the genes, but several other trees were recovered for between 10 and 15% of the genes. Similar results were recently obtained for a number of species of *Drosophila*, where trees showing incongruent phylogenies were found to be clustered across the genome, leading Pollard et al. [21] to suggest incomplete lineage sorting as the likely cause for these observations. It is not known to what degree the genes having incongruent trees are clustered in the genome, but the results suggest that incomplete lineage sorting could be quite common in *Populus*. This is not surprising, since the radiation of the genus *Populus *appear to be relatively recent, within the last 5 Myr [18]. Since the generation time in *Populus *is quite long (> 15 yr), the time since the radiation of the genus, in generations, is rather low.

### Substitution rates at synonymous and non-synonymous sites

Initially I tested for rate heterogeneity among lineages using only synonymous sites by contrasting a model assuming a single molecular clock for the entire phylogeny with a model assuming no clock. These analyses show overwhelming support for a model without a clock, (2Δ_*L *_= 475.7, df = 4, *p *< 0.001), suggesting that the molecular clock hypothesis of a constant substitution rate across the genus *Populus *can be rejected. I then used relative rate tests [22] to compare substitution rates as synonymous sites between pairs of lineages. These tests show significant rate heterogeneities between *P. deltoides *and *P. trichocarpa *(*X*^2 ^= 4.40, *p *= 0.036) and between the *P. nigra *and the *P. tremula*-*P. euphratica *lineages (*X*^2 ^= 103.6, *p *< 0.001). To test for lineage specific effects on synonymous and non-synonymous substitution rates I contrasted a model with a single *d*_*N*_/*d*_*S *_ratio (one-ratio model) with a model allowing for different *d*_*N*_/*d*_*S *_values across branches of the phylogenetic tree (free-ratio model). In general, the log-likelihood values were greater for the free-ratio model than for the one-ratio model and in six of the 158 genes this difference is statistically significant at *p *< 0.05, suggesting significant heterogeneity in selective constraint at these genes. However, none of the genes remain significant after multiple test correction is applied. There was, however, evidence for significant heterogeneity in selective constraint in the concatenated data set (2Δ_*L *_= 13.84, df = 7, *p *= 0.019). The median *d*_*N*_/*d*_*S *_ratio was lowest in *P. trichocarpa *(0.132) and highest in *P. nigra *(0.232) (see also Figure 3 for *d*_*N*_/*d*_*S *_ratios estimated from the concatenated data set). The internal branches leading to the *P. tremula*-*P. euphratica *lineages and the *P. tremula*-*P. euphratica*-*P. nigra *lineages have substantially greater *d*_*N*_/*d*_*S *_ratios (0.316 and 0.349, respectively). However, these branches are very short and *d*_*N*_/*d*_*S *_ratios are subject to a large degree of uncertainty. Nevertheless, all *d*_*N*_/*d*_*S *_ratios are substantially lower than one, suggesting strong purifying selection across all genes.

I also used likelihood ratio tests to test for the presence of heterogeneous selection pressures among sites within individual genes. The null model (M0 [23]), assumes a single *d*_*N*_/*d*_*S *_ratio across sites which is calculated from the data. This model is contrasted with two alternative models; M1a [24], which assumes two classes of sites, one class which is neutral *d*_*N*_/*d*_*S *_= 1 and one class where 0 <*d*_*N*_/*d*_*S *_< 1, and model M2a that in addition to the two classes defined for model M1a allows for a third class of sites with *d*_*N*_/*d*_*S *_> 1 [24]. A total of 14 genes show evidence for heterogeneous selection pressures across codons as evidenced by likelihood ratio tests that were significant at a nominal *p*-value of 0.05. Again none of these tests are significant after multiple test correction. However, this test is known to have low power to detect the action of positive selection when few, relatively closely related sequences are compared in each test, so the results should be interpreted with caution [24]. The concatenated data set provides a more robust test of heterogeneous selection pressures across sites. Here model M1a provides a substantially greater fit to the data, compared to model M0 (2Δ_*L *_= 422, *p *< 0.001). Furthermore, model M2a is significantly better than model M1a (2Δ_*L *_= 40.9, *p *< 0.001), conclusively demonstrating that there is heterogeneity in selection pressures among sites. Under model M2a, the majority of sites (78.3%) are under strong purifying selection (*d*_*N*_/*d*_*S *_≈ 0) and another large class of sites are evolving neutrally (20.2% of sites with *d*_*N*_/*d*_*S *_= 1). Finally, a small fraction of sites (1.4%) appear to be evolving under the action of relatively strong positive selection (*d*_*N*_/*d*_*S *_= 4.2). These sites appear to occur randomly across the genes, with no apparent clustering.

### Changes in codon usage

Ancestral and derived codons were identified across a total of 23787 codons from 158 genes using maximum likelihood methods. A total of 2037 synonymous and 1461 non-synonymous changes were recorded across all lineages (Table 2). The low levels of sequence divergence ensured that ancestral codons could be identified with high degree of certainty and in all cases did the most likely ancestral codon have a probability that exceeded 95%. The estimated number of synonymous and non-synonymous changes in each lineage of the phylogeny are summarized in Table 2. Following Akashi et al. [9], synonymous substitutions were further classified into changes that occurred from preferred to unpreferred codons (*P *→ *U*) or from unpreferred to preferred codons (*U *→ *P*) and deviations from equilibrium codon usage was scored as *d*_*UP*-*PU *_= (*UP *- *PU*)/(*UP *+ *PU*) [9]. Preferred and unpreferred codons were taken from the ΔRSCU analysis described above.

**Table 2:** Lineage-specific substitutions at synonymous (S) and non-synonymous (NS) sites, preferred and unpreferred codons (*U *→ *P *and *P *→ *U*) and A/T and mutations (*W *→ *S *and *S *→ *W*).

Species	S	NS	*P *→ *U*	*U *→ *P*	*G*^*a*^	*d*_*UP*-*PU*_	*S *→ *W*	*W *→ *S*	*G*^*b*^	*d*_*WS*-*SW*_
Pd	292	203	72	102	5.17*	0.172	89	75	1.20	0.085
Pe	561	392	201	181	1.05	-0.052	162	152	0.32	0.032
Pn	282	212	87	84	0.05	-0.018	87	87	0.0	0.000
Pta	548	386	123	227	30.9***	0.297	120	137	1.12	0.066
Ptr	202	120	73	64	0.59	-0.066	36	53	3.24	-0.191
Pd/Ptr	17	49	4	7	0.82	0.273	18	20	0.11	-0.053
Pe/Pta	81	78	33	21	2.67	-0.222	4	12	4.0*	-0.050
Pe/Pta/Pn	54	21	11	6.23*	1.26	0.333	26	31	0.44	-0.087
Total	2037	1461	604	708	8.24**	0.079	559	550	0.07	-0.081

^*a *^Heterogeneity of *P *→ *U *and *U *→ *P *mutations in each lineage

^*b *^Heterogeneity of *S *→ *W *and *W *→ *S *mutations in each lineage

*U *→ *P *mutations shows as slight, but significant excess across the entire phylogeny, suggesting that codon usage has not reached an equilibrium in *Populus*. The excess of *U *→ *P *mutations can, however, largely be explained by the excess of *U *→ *P *substitutions in the *P. tremula *and the *P. deltoides *lineages (Table 2). Both of these lineages do show a consistent excess of *U *→ *P *mutations across loci (*p *= 0.013 and *p *< 0.001 for *P. deltoides *and *P. tremula*, respectively). The remaining lineages appear to have reached a stationary distribution of codon usage and show no significant deviation from an equal number of *P *→ *U *and *U *→ *P *mutations. Taken together, the changes observed in the *P. tremula *and *P. deltoides *lineages suggest that selection on synonymous codon usage codon bias has increased in these two lineages. The degree of codon bias, measured using *F*_*op*_, is higher in *P. tremula *that in both the most closely related species (*P. euphratica*) or in the inferred ancestor, consistent with the observed increase in selection on codon usage. Although these differences are small (mean $\Delta_{F_{op}}$ = 0.0072 and 0.0065 across loci, respectively) they are consistent and highly significant across loci (Wilcoxon signed-ranks test *p *< 0.001 in both cases). Comparisons of *F*_*op *_for *P. deltoides *with either *P. trichocarpa *or their most recent common ancestor are not significant however.

There is also a significant negative correlation between *d*_*UP*-*PU *_and *F*_*op *_across loci over the entire phylogeny (*ρ *= -0.234, *p *= 0.003), as would be expected if selection is shaping synonymous codon usage [12]. I did not detect any correlations between *d*_*S *_and codon bias within any of the lineages. Although codon bias is expected to be negatively correlated to *d*_*S *_when selection on codon bias is strong, theory shows that weak selection (*N s *< 1) actually has little effect on synonymous substitution rates [25].

### Estimating selection on synonymous codons

The full model of Nielsen et al. [10] allows for estimation of independent mutation rates, *d*_*N*_/*d*_*S *_ratios and selection coefficients at synonymous sites in all lineages (Table 3, Figure 3). With 5 species there are 5 × 2 - 2 = 8 branches to estimate these parameters for. The full model outperforms both models assuming strand symmetry, i.e. where mutation rates from nucleotide *i *to *j *is equal the mutation rates from *j *to *i *or models assuming the same mutation rate matrix in all lineages (Table 3). I calculated the expected equilibrium GC content (*π*_*GC*_) from the stationary distribution of nucleotide frequencies obtained from the first normalized eigenvector of the mutation matrix estimated from the data. In all species, except *P. trichocarpa*, the predicted *π*_*GC *_is substantially larger than the observed values (range of predicted *π*_*GC*_: 51.4-62.8%, range of observed *π*_*GC*_: 46.3-46.6%). For *P. trichocarpa *the predicted *π*_*GC *_= 46.7% vs. observed *π*_*GC *_= 46.3%. However, small lineage-specific differences in the estimated mutation rates can translate in to substantial differences in the expected *π*_*GC *_for these lineages, so these differences should be interpreted with caution. When the complete data set is used to estimate a single mutation matrix the expected *π*_*GC *_is substantially closer to the observed values (expected *π*_*GC *_= 43.0%, observed *π*_*GC *_= 46.3%).

**Table 3:** Estimates of selection on synonymous codons for the concatenated sequence data.

Test	log *L*	$2\Delta_{L}^{a}$	df	*p*
Full model	-121729.0	-	-	-
Symmetric mutation rates	-122177.1	896.1	24	*p *< 0.0001
Mutation rates equal in all lineages	-122357.5	1257.1	77	*p *< 0.0001
No selection in *P. deltoides*	-121730.5	3.0	1	0.083
No selection in *P. euphratica*	-121729.7	1.4	1	*p *= 0.237
No selection in *P. nigra*	-121729.4	0.8	1	*p *= 0.371
No selection in *P. tremula*	-121744.4	30.9	1	< 0.0001
No selection in *P. trichocarpa*	-121729.2	0.4	1	*p *= 0.527
Selection equal in all lineages	-121818.6	179.3	8	< 0.001

^*a*^Comparison with the full model

The concatenated data, including data from all 158 genes, show significant evidence for weak, but positive selection on synonymous sites only in the *P. tremula *lineage, with the estimated 4*N*_*e*_*s *= 0.720 (Table 3, Figure 3). The selection coefficient in *P. deltoides *is also marginally significant (*p *= 0.083, Table 3), but with selection only about half as strong as in *P. tremula *(4*N*_*e*_*s *= 0.330).

The gene-specific analyses identify a total of 21 genes that show significant evidence for selection on synonymous codon usage in *P. tremula *at a false discovery rate [26] of 5%. For genes with evidence for significant selection in *P. tremula*, 16 have estimates of *S *> 0 and 5 have *S *< 0. This suggests that although selection on synonymous codon usage favors preferred codons in the majority of cases, there are genes for which there is active selection against preferred codons. This mirror results from *Drosophila*, where the majority of genes show evidence for selection favoring preferred codons but where a few genes show significantly negative estimates of 4*N*_*e*_*s *[27].

### Changes in base composition across lineages

Although the changes in codon usage observed in the *P. tremula *lineage are consistent with an increase in the strength of natural selection acting on codon usage, an alternative explanation is a change in base composition driven by changes in mutational biases. One way to test this assumption is to compare mutational changes from A/T to G/C and vice versa across the phylogeny and in the individual lineages. Since preferred synonymous codons predominantly end with G/C (Figure 1), synonymous sites cannot be used for such comparisons as it would be difficult to disentangle mutational biases from natural selection on codon usage. Ideally, non-coding regions in the vicinity of each gene could be used for such a comparison, since this would account for chromosome-wide differences in base composition. However, since the sequences used for the current analyses are derived from EST projects, non-coding sequences are not available. I have therefore used non-synonymous mutations to infer mutational changes in different lineages.

Following Akashi et al. [9], I classified mutations as *S *→ *W *(strong [G/C] to weak [A/T], following the standard DNA ambiguity code) or *W *→ *S *(weak to strong) across lineages and deviations from equilibrium base composition were quantified as *d*_*WS*-*SW *_= (*WS *- *SW*)/(*WS *+ *SW*). The data show that there are no significant departures from an equilibrium base composition, either across the entire phylogeny or within any of the individual lineages (Table 2). There is also no correlation between *d*_*UP*-*PU *_and *d*_*WS*-*SW *_across loci (*ρ *= 0.112, *p *= 0.16) or between *d*_*WS*-*SW *_and *F*_*op *_(*ρ *= -0.013, *p *= 0.86).

## Discussion

Selection on synonymous codon usage appears to be common among higher eukaryotes [2,3] but the efficacy of such selection is usually thought to be lower in species with lower effective population sizes [4]. Nevertheless, even long-lived species like *Populus *show ample evidence for selection having shaped synonymous codon usage.

Optimal codons appear to be largely the same across the five species used in this analysis, and most of the differences between species can be explained by codons with low ΔRSCU values, where the statistical power to detect changes in codon usage between low and high expression genes may be low. Also, the ΔRSCU analyses are based on publicly available EST-collections and the sets of genes used for the different species are likely very different thereby contributing to the slightly different sets of optimal codons identified in the different species (Figure 1). The codons identified as optimal in this study also correspond well with optimal codons identified in an earlier study of codon bias in *P. tremula *where optimal codons were identified using substantially less data and slightly different methods [15]. The 11 codons identified as optimal in *P. tremula *[15] actually form a subset of 25 optimal codons identified in the ΔRSCU analyses. The estimates of $\bar{\Delta {\text{RSCU}}_{+}}$ for the different species, which provides rough estimates of the overall strength of selection for preferred codons in the different species, is similar to what has been previously observed in *Arabidopsis*, but is lower than observations from either *Drosophila *or *Caenorhabditis *[14,28]. I have used EST data as a proxy for gene expression in this study and this is known to be associated with a lot of problems, like relatively shallow and biased library coverage [29]. However studies that have estimated codon bias parameters using expression profiles from either EST data or other methods, such as MPSS or microarrays, have yielded similar results despite the uncertainties associated with the EST data [28,30,31]. Codon usage bias can in principle be driven by natural selection or by mutational pressure and/or gene conversion [2,7]. Although both of these forces are likely operating, there are unequivocal signs of natural selection having shaped codon usage biases in *Populus*. There are strong positive correlations between gene expression and the degree of codon bias across individual genes in all five species (Table 1). The gene expression-codon bias correlations are independent of base composition differences (Figure 2), indicating that natural selection, operating through translational efficiency or accuracy, has been a significant force shaping synonymous codon usage in *Populus*. The lack of association between *d*_*S *_and codon bias suggest that selection on synonymous codon usage is strong enough to influence rates of synonymous divergence among species. A significant correlation with *d*_*S *_is only expected when natural selection on codon usage is strong. When selection on codon bias is weak, theory shows that it has only small effects on the rate of synonymous substitutions even if the effects on codon usage can be significant [25].

Interestingly, gene length appears to have virtually no effect on codon bias, except in *P. trichocarpa *(Figure 2). Earlier studies in *P. tremula *have suggested a weak, but significantly negative effect of gene length on codon bias [15] and negative correlations have been found in other eukaryotes as well [28]. Several reasons can explain the lack of a correlation between gene length and codon bias in the present study. First, there might be genuine differences between species in the effect of gene length. Cutter et al. [14] found that for some species of *Caenorhabitis *gene length had a significant effect on codon bias whereas for other species no such effect were found, suggesting that the effects of gene length might differ even between closely related species. Alternatively, different sets of genes were used for the different species and it is conceivable that this could explain why an effect of gene length is lacking in some species. However, the number of genes used is quite large for all species (Table 1), so if gene length has an effect the sample sizes are likely large enough that it would be detected. Finally, gene lengths were taken from the annotated genome of *P. trichocarpa *but were assumed to be representative of the corresponding gene from the remaining species. It is possible, although perhaps unlikely, that gene lengths differ substantially between species. Nevertheless, the effects of gene length on codon bias clearly deserves further attention.

At first impression, codon usage does not appear to have reached an equilibrium in *Populus *as there was an excess of *U *→ *P *substitutions across the entire phylogeny (Table 2). However, the excess of *U *→ *P *substitutions could largely be explained by significant excesses in two different lineages, *P. deltoides *and *P. tremula*, with the remaining lineages showing no deviations from an equal numbers of *P *→ *U *and *U *→ *P *substitutions(Table 2). In addition, the maximum-likelihood estimates of the selection coefficient acting on synonymous codons are positive in both of these lineages (4*N*_*e*_*s *= 0.330 in *P. deltoides *and 4*N*_*e*_*s *= 0.720 in *P. tremula*).

The 4*N*_*e *_estimate for *P. tremula *is significantly greater than zero whereas the estimate for *P. deltoides *is approaching significance (Table 3). The estimate of selection in *P. tremula *(S = 0.720) is only slightly lower than estimates of selection from *Drosophila simulans *and *D. yakuba *[10], two species for which there are abundant evidence for selection on synonymous codon usage [9,10]. In addition, *P. tremula *has also significantly higher *F*_*op *_than both the most closely related species (*P. euphratica*) or their common ancestor, supporting an increase in the degree of codon bias as a result of ongoing selection for preferred codons in *P. tremula*.

For the remaining lineages of the phylogeny the maximum-likelihood estimates of 4*N*_*e*_*s *are either close to zero or even negative, although none of these estimates are significantly different from zero (Table 3). These results suggest that the strength of selection acting on codon usage has increased independently in two different lineages in the genus *Populus*, with natural selection favoring preferred codons in *P. tremula *and *P. deltoides*. Despite lacking evidence for selection on preferred codons, the remaining species show signs of past selection on codon usage as evidenced by the positive association between gene expression and codon bias discussed above. Finally there is no evidence for changes in base composition across lineages (Table 3), although these conclusions are based on changes at non-synonymous sites. There are significant differences in the GC content of coding regions and surrounding non-coding regions in *P. tremula *[15] but also no apparent correlation between GC contents of coding and non-coding regions, suggesting that non-synonymous sites might provide an accurate picture of the patterns of mutations in coding regions in *Populus*.

What accounts for the increase in the strength of selection acting on synonymous codons in *P. tremula *(and in *P. deltoides*)? One thing that distinguish *P. tremula *from many other species of *Populus *is its very wide distribution, ranging throughout Eurasia, from Western Europe to Eastern Asia. Therefore based only on the distribution range, *P. tremula *likely has a substantially greater effective population size than other species of *Populus*, that in many cases have both geographically more restricted ranges and more fragmented distributions where they do occur [18]. It is interesting to note that *P. deltoides *also have a relatively wide distribution range [18], suggesting that the *N*_*e *_could be relative large also in *P. deltoides*. Sequence-based estimates of the effective population size of *P. tremula *suggest that *N*_*e *_is at least 10^5 ^[32]. This estimate of *N*_*e *_is heavily dependent on the genome-wide mutation rate in *Populus *and this has not been characterized in great detail [33]. However, estimates of *N*_*e *_are lacking for other species of *Populus*, and the conclusions regarding the relative population sizes of the different species are clearly tentative.

In other species, population size in known to have a large effect on the strength of selection acting on preferred codons. As mentioned above, the effective population size appears to have been reduced in *D. melanogaster *and this have resulted in reduced selection on synonymous codons at least when compared to the closely related species *D. simulans *[8-10]. A similar picture is emerging from data on synonymous codon usage in nematodes where species that have evolved self-fertilization and/or have a parasitic life-style have experienced a reduction in *N*_*e *_which translates into lower selection on codon bias [14,16]. These results suggest that changes in effective population size are perhaps the most important parameter explaining differences in codon bias between closely related species. Interestingly, selection on codon usage does not differ dramatically between *Arabidopsis thaliana *and *A. lyrata*, despite the the former bing highly selfing and the latter obligately out-crossing. This mirrors data on rates of protein evolution which also does not differ between the two species and which suggest that the effective population sizes are not dramatically between *A. thaliana *and *A. lyrata *[34].

## Conclusion

This study conclusively demonstrates that natural selection on synonymous codon usage is occurring also in a long-lived, perennial plant species like *Populus*. Data on synonymous codon usage suggest both ancient selection on synonymous codon usage in the genus *Populus *and current selection in *P. tremula*. The data also show that there has been a shift in the strength of selection acting on preferred codons in the *P. tremula *lineage and possibly also in *P. deltoides*. The analyses of the present data make shifts in mutations rates among species unlikely, suggesting that increases in the effective population size in these lineages can explain the observed increase in selection on synonymous mutations.

## Methods

### EST analysis

I downloaded all available ESTs for five different species of Populus, *P. deltoides *(14661 ESTs), *P. euphratica *(13905), *P. nigra *(51361), *P. tremula *(37313) and *P. trichocarpa *(89943) from PlantGDB [35]. I also downloaded the corresponding PlantGDB-assembled Unique Transcripts (PUT) for *P. deltoides*, *P. euphratica*, *P. nigra *and *P. tremula*. These PUTs are unique transcripts assembled from all mRNA sequences for a given species available in public databases and have been trimmed to remove bacterial contamination, repetitive sequences and polyA tails. For the remainder of this paper I will refer to these PUTs as genes, recognizing that these PUTs do, for the most part, not correspond to full-length transcripts. I also downloaded all predicted gene models for *P. trichocarpa *from the publicly available genome sequence at http://genome.jgi-psf.org/Poptr1_1/Poptr1_1.home.html. Reading frames of all genes were checked, and corrected, for sequencing errors using FrameD [36]. Pairwise comparisons were then performed between all genes within species using BLASTN and redundant sequences as well as sequences from gene families with very similar paralogous copies (> 85% sequence similarity) were removed. Finally, sequences shorter than 300 bps were also excluded. The final numbers of unique genes per species are listed in Table 1.

Expression profiles for all genes were obtained by all-against-all BLASTN searches of genes and the complete EST data for each species separately. Gene-EST alignments were required to show at least a 90% identity across 100 bp to be recorded as a match and the number of BLASTN hits were used as a proxy for expression levels. I partitioned the gene expression data for each species into two classes, genes with a single EST hit and genes which had EST hit numbers that were equal or greater than the species specific 90th percentile [14,28]. I then calculated relative synonymous codon usage (RSCU) for all genes in each species and expression category separately using the codonw (version 1.4.2 http://codonw.sourceforge.net/). I then identified codons which are over-represented in highly expressed genes by comparing differences in ΔRSCU between high and low expression genes (ΔRSCU) using *t*-tests using R [37]. Codons showing significantly higher levels of ΔRSCU in high expression genes were taken as optimal codons for the species in question. Finally, following Cutter et al. [14], overall indices of codon usage for each of the five different species were calculated as the average of all positive ΔRSCU values, $\bar{\Delta {\text{RSCU}}_{+}}$ (Table 1).

Optimal codons identified from the ΔRSCU analyses were used to calculate the frequency of optimal codons (*F*_*op*_) [38] and the GC content of the coding region using codonw http://codonw.sourceforge.net. Each gene was also associated with the most likely orthologous gene from the *P. trichocarpa *genome sequence using BLAST. The lengths of the corresponding full-length sequences from *P. trichocarpa *were used in combination with gene expression (EST hit count) and base composition (GC content) to estimate the amount of variation in *F*_*op *_that the variables and their first order interactions explain [14]. As pointed out by Cutter et al. [14], a significant positive effect of gene expression is consistent with selection having shaped optimal codon usage.

### Identification of orthologous genes and calculations of substitution rates

Putative one-to-one orthologous genes were identified from the five different species using the program orthoMCL, using default parameters [17]. Groups of putatively orthologous sequences were aligned using ClustalW [39]. All identified genes were also concatenated within species and used to construct the most likely phylogenetic tree using baseml from the PAML package. The baseml analyses used the HKY85 substitution model with rate variation among sites and with the transition:transversion ratio estimated from the data (HKY85+Γ). The resulting tree was rooted based on results from phylogenetic analyses of the entire genus *Populus *[20].

Synonymous and non-synonymous substitutions for each gene and each lineage of the phylogeny were calculated using the codeml program from PAML [40] based on the phylogenetic tree obtained from the concatenated data set. The model assumed different *d*_*N*_/*d*_*S *_ratios for all branches in the phylogeny (i.e model = 1) and with codon frequencies calculated average nucleotide frequencies at the three codon positions (*F*3*x*4).

To test for evidence of heterogeneous selection acting on codons, codeml from PAML was used to fit a null model with a single *d*_*N*_/*d*_*S *_ration (model M0 [23]) and two models that allow the *d*_*N*_/*d*_*S *_ratio to vary between codons (models M1a and M2a [24]). A gene was assumed to be under positive selection if a likelihood ratio test, comparing models M1a and M2a, was significant at *p *< 0.05. As *d*_*S *_did not show any association with codon bias it was not necessary to correct *d*_*S *_for possible effects of selection at synonymous sites [41].

### Ancestral sequence reconstruction

Ancestral sequences at internal nodes of the phylogeny were reconstructed using baseml from the PAML package [40]. baseml use a defined substitution model and a phylogenetic tree to estimate branch lengths and to assign posterior probabilities to different ancestral nucleotides at internal nodes of the phylogenetic tree. The ancestral reconstruction also used the HKY85+Γ nucleotide substitution model. Probabilities of ancestral codons were calculated as the product of probabilities of ancestral nucleotides at the three different nucleotide positions [9].

For codons that differ at more than a single nucleotide position, there are multiple alternative paths between ancestral and derived codons. I used the method of Akashi et al. [9] which weight different paths equally. With low levels of sequence divergence, as in this data set, this method give results similar to methods that weight paths by the number of synonymous to non-synonymous substitutions [9]. However this method has a much lower computational demand. Following Akashi et al. [9] multiple hits were ignored, again motivated by the low levels of sequence divergence in the data set (*d*_*S *_< 5 – 7%). Once the ancestral sequences of the internal nodes of the tree had been obtained, lineage-specific synonymous and non-synonymous substitutions were calculated. In addition, synonymous substitutions were classified according to the putative fitness effects of the ancestral and derived codons. Codon preferences for all species were assumed to follow the preference table established from the ΔRSCU analyses described above. Mutations between codon preference classes were classified as unpreferred to preferred (*P *→ *U*) or preferred to unpreferred (*P *→ *U*) depending on the state of the ancestral and derived codons. Mutations within codon classes (*U *→ *U *and *P *→ *P*) were also scored.

If codon usage is at equilibrium, an equal number of *U *→ *P *and *P *→ *U *mutations are expected. To test this assumption I applied a goodness-of-fit test to the number of *P *→ *U *and *U *→ *P *mutation in each lineage of the phylogeny.

### Estimating selection at synonymous sites

To estimate the strength of selection acting on alternative codons in different lineages of the *Populus *phylogeny I used the method of Nielsen et al. [10]. Briefly, the method of Nielsen et al. [10] extends codon-based likelihood methods [42] with a parameter that allow for selection on synonymous codon usage. The method estimate the scaled coefficient of selection (*S *= 4*N*_*e*_*s*) acting on mutations from unpreferred to preferred codons, with *S *> 0 indicating selection favoring the preferred state and *S *< 0 indicating selection against the preferred state. In addition to estimating *S*, the method also provides lineage specific estimates of the ratio of non-synonymous to synonymous substitution rates (*ω *= *d*_*N*_/*d*_*S*_) and parameters of the mutation matrix [10]. The input tree and branch lengths were taken from the PAML analyses described above.

Mutation rates were initially estimated using a data set consisting of concatenated sequences from all loci. Technically it is difficult to accurately estimate the mutation rate parameters on either of the lineages surrounding the root, because the placement of the root in the tree not uniquely determined in the data set. Mutation rate estimates for these lineages are therefore only approximate. Mutation rates estimated from the concatenated sequence data were then used in gene-specific analyses to reduce computational time.

To test for selection on synonymous sites, using either the concatenated data sets or individually for all loci, I separately fit two models. The first model has *S *as a free parameter and therefore allows for lineage specific estimation of *S*. The alternative model constrains *S *to zero on a specific lineage of interest and therefore assumes that synonymous sites are neutral along that lineage. The two models are nested and can be compared using a likelihood ratio test, where twice the difference in the likelihood of the two models (Δ_log*L *_= log(*L*_2_) - log(*L*_1_)) is assumed to follow a *χ*^2^-distribution with degrees of freedom equal to the difference in number of parameters between the two models (*n*_*p*(2) _- *n*_*p*(1)_).

## Authors' contributions

PKI designed the study, performed all statistical analyses and wrote the paper.
